# Safety and adverse events associated with dexmedetomidine for sedation in adult ICU patients: a systematic review and meta-analysis

**DOI:** 10.3389/fmed.2025.1677955

**Published:** 2025-11-07

**Authors:** Yingwei Ding, Xiaojun Wang, Xiuhua Li, Jiantao He, Xusheng Teng, Gang Chen

**Affiliations:** Department of Emergency Medicine, Jinhua Municipal Central Hospital, Affiliated Jinhua Hospital, Zhejiang University School of Medicine, Jinhua, China

**Keywords:** dexmedetomidine, intensive care unit, sedation, safety, adverse events

## Abstract

**Background:**

Dexmedetomidine (DEX) is increasingly used for sedation in critically ill adults due to its favorable pharmacokinetic profile and potential benefits over traditional sedatives. However, concerns persist regarding its cardiovascular safety. This meta-analysis comprehensively evaluates the incidence and nature of adverse events associated with DEX sedation in adult intensive care unit (ICU) patients.

**Methods:**

We conducted a systematic review and meta-analysis according to PRISMA 2020 guidelines. A meta-analysis search of PubMed, Embase, and Cochrane Library was conducted from database inception to June 18, 2025 for randomized controlled trials (RCTs), prospective/retrospective cohort studies, or descriptive studies with a comparator group, reporting safety outcomes in adults receiving DEX for ICU sedation. Primary outcomes were hemodynamic adverse events including hypotension, bradycardia, tachycardia. Data were pooled using fixed-effects models, calculating odds ratios (ORs) with 95% confidence intervals (CIs). Heterogeneity was assessed using I^2^ statistics. Risk of bias was evaluated using Cochrane RoB 2.0 for RCTs and Newcastle-Ottawa Scale (NOS) for observational studies. GRADE assessed evidence certainty.

**Results:**

Ten studies (7 randomized controlled trials, 3 cohorts; total *n* = 1,456 patients) were included. Meta-analysis demonstrated a significantly increased risk of bradycardia with dexmedetomidine versus controls (9 studies, *n* = 1,590; pooled OR = 2.38, 95% CI [1.77, 3.21], *p* < 0.00001; I^2^ = 0%; high certainty evidence). No significant increase in overall hypotension risk was observed (9 studies, *n* = 1,422; OR = 1.15, 95% CI [0.91, 1.47], *p* = 0.25; I^2^ = 44%; moderate certainty), though subgroup analyses indicated elevated risks in vulnerable populations. A modest but significant increase in tachycardia risk was found (4 studies, *n* = 1,084; OR = 1.38, 95% CI [1.03, 1.83], *p* = 0.03), with substantial heterogeneity (I^2^ = 93%; low certainty) suggesting context-dependent effects. Risk of bias was generally low for RCTs, while observational studies demonstrated good quality but limited confounding adjustment.

**Conclusion:**

Dexmedetomidine use for ICU sedation is consistently associated with a significantly increased risk of bradycardia. While the overall risk of hypotension was not significantly elevated, specific patient populations may be vulnerable. Tachycardia risk appears modest but highly variable. These findings underscore the necessity for careful patient selection, continuous hemodynamic monitoring particularly heart rate, and cautious titration when using DEX in critically ill adults. Future research should focus on high-risk subgroups and standardize adverse event definitions.

## 1 Introduction

Sedation in the intensive care unit (ICU) is essential for patient comfort, facilitation of mechanical ventilation, and reduction of stress responses, yet traditional sedatives such as benzodiazepines and propofol carry risks of prolonged ventilation, delirium, and respiratory depression (1). These adverse outcomes have prompted investigation into alternative agents that may offer effective sedation with a more favorable safety profile. Dexmedetomidine is a selective α_2_-adrenoceptor agonist that produces sedation resembling natural sleep, with the ability to provide cooperative sedation and some analgesia without significant respiratory depression (2). Its dose-dependent sympatholytic action sets it apart from GABA-ergic sedatives and gives rise to a distinct pattern of hemodynamic effects (3).

Clinical trials comparing dexmedetomidine with midazolam or propofol have demonstrated comparable efficacy in achieving light to moderate sedation, with the added benefits of reduced duration of mechanical ventilation and improved patient interaction. For example, the MIDEX-PRODEX trials found that dexmedetomidine was non-inferior to midazolam and propofol in maintaining target sedation levels and was associated with shorter ventilation times compared to midazolam (4). Unlike midazolam dexmedetomidine does not have any active metabolites and this may be responsible for the shorter ventilation times. Similarly, the MENDS trial showed that dexmedetomidine reduced the prevalence of acute brain dysfunction compared to lorazepam (5). In addition, some studies have reported that dexmedetomidine administration in critically ill adults may reduce the incidence of delirium during ICU stays (6, 7). Although opioids have been traditionally used in the ICU for their sedative and analgesic properties, they carry a risk of significant side effects, including respiratory depression, delirium, gastrointestinal issues such as nausea, vomiting, ileus, and autonomic effects such as urinary retention, pruritus, and prolonged dependence on positive pressure ventilation (8–11). Dexmedetomidine offers a distinctly different adverse effect profile and is not typically associated with these complications. Taken together, the findings from these studies support the favorable profile of dexmedetomidine in critically ill populations.

Despite these advantages, dexmedetomidine’s safety profile warrants close attention. Hypotension and bradycardia are the most frequently reported adverse events, reflecting its sympatholytic action (3). In large randomized studies, patients receiving dexmedetomidine treatment were more likely to develop bradycardia and hypotension (2, 4). The SPICE III trial further highlighted an increased incidence of serious adverse events, including severe bradyarrhythmia, in the dexmedetomidine arm (3). Study definitions and reporting of adverse events vary, and most individual trials are under-powered to characterize infrequent but serious complications. To date, a comprehensive synthesis of the safety profile of dexmedetomidine remains lacking, which limits our ability to balance its sedative benefits with potential risks and to inform individualized sedation strategies and clinical monitoring standards. Accordingly, this meta-analysis seeks to evaluate the incidence and nature of dexmedetomidine-related adverse events in adult ICU patients, in order to inform safer and more evidence-based sedation practices.

## 2 Methods

### 2.1 Study design

This systematic review and meta-analysis was conducted in accordance with the Preferred Reporting Items for Systematic Reviews and Meta-Analyses (PRISMA) 2020 guidelines (12). This study was conducted in strict accordance with the pre-defined protocol, with no deviations (Supplementary Figure 1).

### 2.2 Eligibility criteria

#### 2.2.1 Population and Intervention

We included studies enrolling adult patients (≥18 years) admitted to intensive care units (ICUs), regardless of underlying illness or ICU subtype (medical, surgical, trauma, or mixed). Studies conducted in non-ICU environments (e.g., post-anesthesia care units or procedural sedation settings) or focusing on pediatric populations were excluded. Eligible studies assessed the use of dexmedetomidine for sedation in ICU patients, either as monotherapy or in combination with other sedatives or analgesics. Dexmedetomidine used primarily for other purposes (e.g., blood pressure control or delirium prevention) without sedation context were excluded. Comparator groups included standard sedatives such as propofol, midazolam, or other agents, placebo, or standard care without dexmedetomidine. The primary outcomes were safety-related adverse events, including bradycardia, hypotension, tachycardia, respiratory depression, and any reported serious adverse events (SAEs).

### 2.3 Study types

We included original clinical studies of the following types: Randomized controlled trials (including open-label designs), Prospective cohort studies, Retrospective cohort studies, Descriptive studies of clinical practice (provided they included a comparison group). We excluded case reports, case series, editorials, letters, conference abstracts, review articles, meta-analyses, and protocols. Only studies reporting at least one eligible adverse event were included.

### 2.4 Information sources and search strategy

We systematically searched PubMed, Embase, and the Cochrane Library from database inception to June 18, 2025. A comprehensive search strategy was developed using a combination of controlled vocabulary (e.g., MeSH, Emtree) and free-text terms related to dexmedetomidine, ICU sedation, and adverse events. The search terms included variations of the following: (“dexmedetomidine” OR “right medetomidine” OR “Precedex”) AND (“intensive care units” OR “ICU” OR “critical care” OR “intensive care”) AND (“sedation”) AND (“adult” OR “aged” OR “18 years”) AND (“adverse effects” OR “adverse event” OR “side effect” OR “toxicity” OR “safety”). We applied filters to exclude the following publication types: case reports, case series, letters, editorials, comments, correspondence, reviews, meta-analyses, conference abstracts, and protocols. Additional eligible studies were identified by manually screening the reference lists of relevant articles and reviews. The detailed search terms in this study by PubMed, Embase, and the Cochrane Library as shown in Supplementary Table 1.

### 2.5 Study selection and data extraction

Two reviewers independently screened titles and abstracts to identify potentially eligible studies. Full-text articles were retrieved and assessed according to the inclusion and exclusion criteria. Disagreements were resolved by discussion or adjudication by a third reviewer. A standardized data extraction form was used to collect information on study characteristics including author, year, country, design, patient demographics, sedation protocol, comparators, dexmedetomidine dosage and duration, and adverse event outcomes. Data were extracted independently by two reviewers, with discrepancies resolved by consensus.

### 2.6 Data synthesis and statistical analysis

Dichotomous outcomes were pooled using odds ratios (OR) with 95% confidence intervals (CIs) via a fixed-effects model. Heterogeneity was assessed using the I^2^ statistic, with I^2^ > 50% indicating substantial heterogeneity. If meta-analysis was not feasible, a narrative synthesis was provided. All statistical analyses, including meta-analyses and forest plot generation, were conducted using Review Manager (RevMan) version 5, developed by the Cochrane Collaboration.

### 2.7 Risk of bias assessment

Risk-of-bias of randomized trials were assessed independently by two investigators using the Cochrane risk-of-bias tool for randomized trials, version 2 (RoB 2) (13). Risk-of-bias of non-randomized studies were evaluated using the Newcastle-Ottawa Scale (NOS), tailored by study design (cohort or descriptive) (14). The certainty of evidence for each outcome was systematically evaluated using the GRADE (Grading of Recommendations Assessment, Development and Evaluation) approach (15). We initially assigned high certainty to evidence from randomized controlled trials (RCTs) and low certainty to observational studies. Through the GRADE assessment process, we evaluated five key domains that could modify the initial certainty rating: (1) risk of bias (assessed using Cochrane RoB 2.0 for RCTs and Newcastle-Ottawa Scale for observational studies); (2) inconsistency (quantified by I^2^ statistics and visual inspection of forest plots); (3) indirectness (considering population, intervention, comparator, and outcome applicability); (4) imprecision (based on sample size and confidence interval width); and (5) publication bias assessed via funnel plots.

## 3 Results

### 3.1 Search and selection

A comprehensive literature search was conducted across multiple databases including PubMed, EMBASE, Cochrane Library from inception to June 2025. The search strategy combined terms for dexmedetomidine (“Dexmedetomidine” OR “Dexmedetomidin*”), ICU populations (“Intensive Care Units” OR “ICU” OR “critical care”), and safety outcomes (“Hypotension” OR “Bradycardia” OR “Tachycardia” OR “Respiratory Depression” OR “Adverse Drug Event” OR “Adverse Event”). Two independent reviewers screened records in three phases: title/abstract screening excluding non-ICU settings and animal studies, full-text assessment requiring RCTs or cohorts with control groups and quantitative safety data, and final inclusion based on complete outcome reporting. From an initial pool of 451 records, 10 studies including 7 RCTs and 3 cohort studies met all eligibility criteria and were included in the final analysis. The selection process followed PRISMA guidelines, with disagreements resolved through consensus or third-party adjudication. This rigorous approach ensured inclusion of high-quality evidence while minimizing selection bias (Supplementary Figure 1).

### 3.2 The characteristics of included studies

The included studies comprised 10 clinical trials, including 7 randomized controlled trials and 3 non-randomized observational studies, involving a total of 1,456 ICU patients across multiple countries and regions, including the United States, China, Japan, Iran, Egypt, Spain, and Australia (Table 1) (2, 16–24). These studies evaluated dexmedetomidine for sedation in various critically ill populations, such as mechanically ventilated patients, septic patients, and elderly patients with delirium. Sample sizes per study ranged from 33 to 366 patients. The mean age of participants varied from 40.55 to 79.46 years, and the proportion of male patients ranged from 41.94 to 86.36%. DEX was primarily administered as monotherapy, although some studies permitted adjunctive sedatives (e.g., midazolam, propofol) based on clinical need. Comparator groups included midazolam, propofol, haloperidol, olanzapine, or standard care. The most commonly reported adverse events were hemodynamic disturbances, including hypotension (reported in 10 studies), bradycardia (9 studies), and tachycardia (4 studies), along with other events such as respiratory depression, nausea, vomiting, and arrhythmias. Study designs included double-blind RCTs, open-label trials, and retrospective cohort studies, providing a comprehensive overview of the safety profile of DEX in adult ICU patients. The detailed information as shown in Table 1.

**Table 1:** Characteristics of the included studies.

Study	Country	Design	Patient type	Sample size (Dex/Ctrl)	Sedation protocol	Control regimen	Mean age (years) Mean ± SD	Male (%)	Adverse events reported
Mokhlesian M, 2025	Iran	A double-blind, randomized trial	Septic shock	24/24	DEX alone	Morphine plus midazolam	60.63 ± 17.27	52.01	Hypotension, bradycardia, respiratory depression, delirium
Ibrahim AM, 2024	Egypt	A randomized, open-label trial	Receive MV	20/20	DEX alone	Propofol	40.55 ± 12.42	60	Hypotension, bradycardia
Liu SB, 2021	China	A retrospective cohort study	Critically ill elderly patients with delirium/without ventilation or surgery	118/145	DEX, when the effect did not reach a satisfactory RASS level added diazepam or midazolam	Olanzapine, when the effect did not reach a satisfactory RASS level added diazepam or midazolam	79.46 ± 5.44	76.81	Hypotension, bradycardia, tachycardia, respiratory depression, hypoxia, coma
Hughes CG, 2021	United state	A double-blind, randomized, controlled trial	Suspected or known infection, and were treated with continuous sedation for invasive mechanical ventilation	214/208	DEX alone	Propofol	59.49 ± 14.09	57.11	Hypotension, bradycardia, tachycardia, severe lactic acidosis, CV SOFA (cardiovascular Sequential Organ Failure Assessment)
Kawazoe Y, 2017	Japan	An open-label, multicenter randomized clinical trial	Sepsis and needed mechanical ventilation	100/101	DEX, minimum propofol/ midazolam were added as needed	Propofol, midazolam	68.49 ± 14.23	63.18	Bradycardia, acute coronary syndrome
Genís C, 2016	Spain	A nonrandomized, controlled trial (quasi-experimental) and unicenter	Nonintubated patients with a diagnosis of agitated delirium	46/86	DEX and haloperidol	Haloperidol	70.94 ± 11.69	86.36	Abnormal corrected for heart rate QT interval, supraventricular/ventricular/arrhythmia, Bradycardia, hypotension
Devlin JW, 2014	United state	A randomized, double-blind, placebo-controlled pilot study	Adults with ARF and within 8 h of starting NIV	16/17	DEX, midazolam was added as needed	Haloperidol, midazolam	64.91 ± 13.06	51.52	Supraventricular tachycardia, hypotension
Huang Z, 2012	China	A randomized, open-label, prospective trial	Acute cardiogenic pulmonary edema and hypoxemia	33/29	DEX alone	Midazolam	64.64 ± 8.28	41.94	Hypotension, bradycardia, delirium, vomiting, gastric aspiration, respiratory infections
Anger KE, 2010	United state	A prospective, descriptive study of clinical practice.	Postoperative mechanically ventilated cardiac surgery patients	28/28	DEX alone	Propofol	66.36 ± 12.29	69.64	Hypotension, bradycardia
Riker RR, 2009	United States, Australia, Brazil, Argentina, New Zealand	Prospective, double-blind, randomized trial	Patients with expected mechanical ventilation for more than 24 h	244/122	DEX alone	Midazolam	61.97 ± 15.49	49.73	Bradycardia, tachycardia, hypotension, hypotension, hyperglycemia, infection

### 3.3 Adverse events comparison

**Figure 1:**
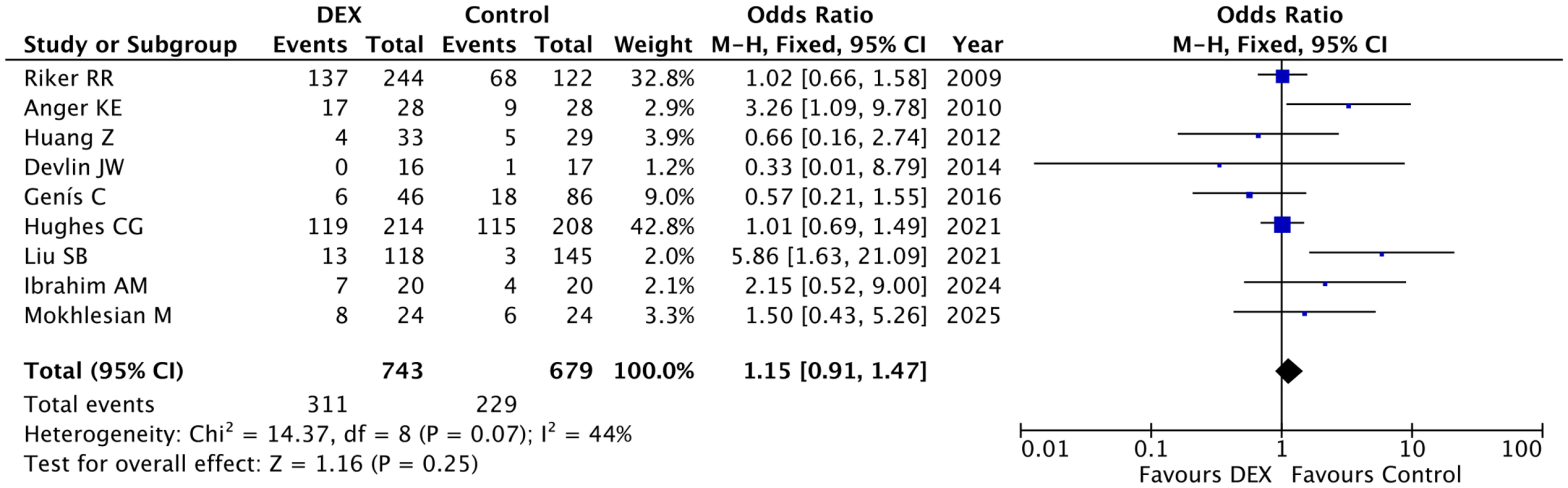
Forest plot of hypotension incidence comparing intervention and control groups. This meta-analysis included nine studies involving a total of 1,422 patients. A fixed-effects model was used. Dexmedetomidine use was not associated with a statistically significant increase in the risk of hypotension compared to control groups (pooled OR = 1.15, 95% CI [0.91, 1.47]). Moderate heterogeneity was observed among studies (I^2^ = 44%, *p* = 0.07).

Hypotension: The meta-analysis of nine studies involving 1,422 patients found no statistically significant increase in hypotension risk with dexmedetomidine compared to control treatments (pooled OR = 1.15, 95% CI [0.91, 1.47], *p* = 0.25), with moderate heterogeneity observed (I^2^ = 44%, *p* = 0.07) (Figure 1). While most studies showed no significant difference in hypotension risk, two smaller trials reported significantly higher odds of hypotension with dexmedetomidine (OR = 3.26 and 5.86, respectively) (18, 24), potentially reflecting dose-dependent effects or high-risk populations. Although the overall analysis suggests dexmedetomidine does not substantially increase hypotension risk, the variability across studies, particularly in smaller trials, indicates that individualized risk assessment remains important, especially in hemodynamically unstable patients. These findings support cautious use of dexmedetomidine with close blood pressure monitoring, particularly during initial dosing, while highlighting the need for further research to clarify dose- and population-specific effects.

**Figure 2:**
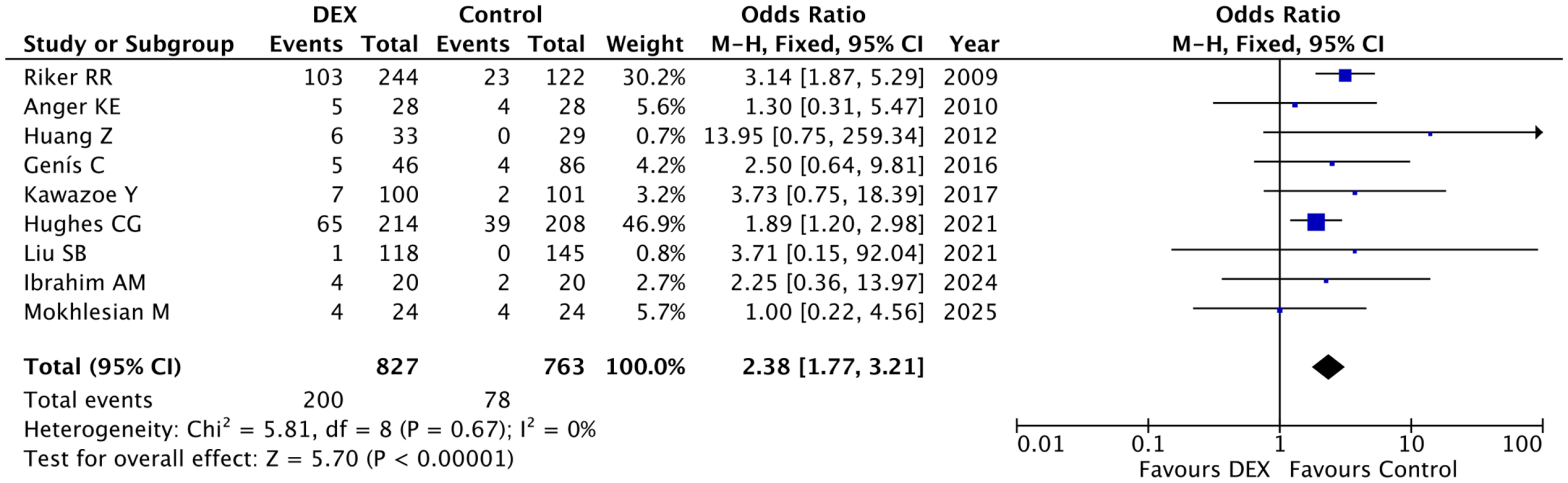
Forest plot of bradycardia incidence in dexmedetomidine versus control groups. A fixed-effects model was applied to analyze data from nine studies involving a total of 1,590 patients. Dexmedetomidine use was associated with a significantly increased risk of bradycardia compared to control treatments (pooled OR = 2.38, 95% CI [1.77, 3.21], *p* < 0.00001), with no significant heterogeneity observed among studies (I^2^ = 0%, *p* = 0.67).

Bradycardia: The meta-analysis of nine studies involving 1,590 patients demonstrated a statistically significant increase in bradycardia risk with dexmedetomidine compared to control treatments (pooled OR = 2.38, 95% CI [1.77, 3.21], *p* < 0.00001), with no observed heterogeneity (I^2^ = 0%, *p* = 0.67) (Figure 2). The largest study (weight = 46.9%) showed a clear increased risk (OR = 1.89, 95% CI [1.20, 2.98]) (19), while the most pronounced effect was observed in Riker RR 2009 (OR = 3.14, 95% CI [1.87, 5.29]) (2). Although some smaller studies did not reach statistical significance due to wide confidence intervals (e.g., Huang Z 2012: OR = 13.95, 95% CI [0.75, 259.34]) (23), the overall consistent direction of effect across all studies strongly suggests that dexmedetomidine use is associated with elevated bradycardia risk. These findings indicate that careful heart rate monitoring and cautious use in patients with pre-existing conduction abnormalities or those receiving concomitant bradycardia-inducing medications are warranted when administering dexmedetomidine in ICU settings.

**Figure 3:**
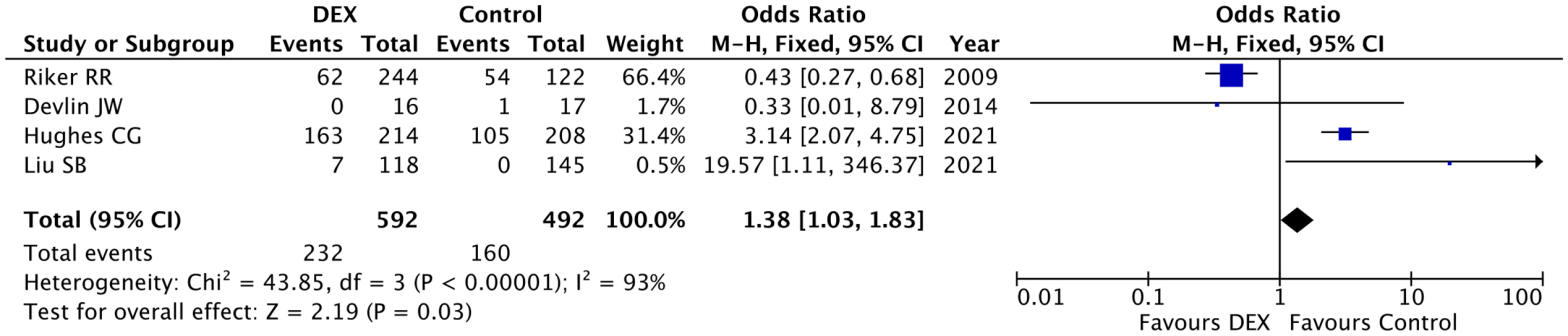
Forest plot of tachycardia incidence associated with dexmedetomidine use versus control. This meta-analysis included four studies with a total of 1,084 patients. A fixed-effects model was used. Dexmedetomidine use was associated with a modest but statistically significant increase in the risk of tachycardia compared to control groups (pooled OR = 1.38, 95% CI [1.03, 1.83], *p* = 0.03). Substantial heterogeneity was observed across studies (I^2^ = 93%, *p* < 0.00001).

Tachycardia: The meta-analysis of four studies (*n* = 1,084 patients) examining tachycardia risk showed a statistically significant increase associated with dexmedetomidine use compared to controls (pooled OR = 1.38, 95% CI [1.03, 1.83], *p* = 0.03), though with substantial heterogeneity (I^2^ = 93%, *p* < 0.00001) (Figure 3). The results demonstrated marked variability across studies: Hughes CG 2021 (weight = 31.4%) reported significantly higher tachycardia risk with dexmedetomidine (OR = 3.14, 95% CI [2.07, 4.75]) (19), while Riker RR 2009 (weight = 66.4%) paradoxically showed a protective effect (OR = 0.43, 95% CI [0.27, 0.68]) (2). Extreme outliers included Liu SB 2021 (OR = 19.57, 95% CI [1.11, 346.37]) with very wide confidence intervals (18), and Devlin JW 2014 reporting zero events in the DEX group (22). The extreme heterogeneity suggests important differences in study populations, dosing regimens, or outcome definitions that require further investigation. These findings indicate that while dexmedetomidine may increase tachycardia risk overall, the effect appears highly context-dependent, necessitating careful patient selection and monitoring, particularly in populations where sympathetic activation might be problematic. The contradictory results between major studies highlight the need for standardized tachycardia definitions and additional research to clarify risk factors.

### 3.4 Quality assessment

The Newcastle-Ottawa Scale (NOS) assessment of the three cohort studies (Liu SB 2021, Genís C 2016, Anger KE 2010) demonstrated high methodological quality, with each study scoring 7 out of 9 points (Table 2) (18, 21, 24). All studies achieved full marks in the Selection domain (4 points), including representativeness of exposed cohorts, appropriate selection of non-exposed cohorts, objective exposure ascertainment, and demonstration of no baseline outcomes. However, in the Comparability domain, all studies received only 1 point as the study identified and reported key baseline confounders (e.g., age, APACHE II score), but no multivariate adjustment or propensity score methods were performed to adjust for confounding. Full points were awarded in the Outcome domain (3 points) for objective outcome assessment, adequate follow-up duration, and complete follow-up. While these scores indicate low risk of bias overall, the limited control for confounding in the Comparability domain suggests potential limitations in causal inference. The NOS results support inclusion of these studies in the analysis, with acknowledgment of inherent observational design limitations in the discussion.

**Table 2:** The Newcastle-Ottawa Scale (NOS) assessment of the three cohort studies (Liu SB 2021, Genís C 2016, Anger KE 2010).

	Selection (4)				Comparability (2)	Outcomes (3)			
Study	Representativeness of exposed cohort (★)	Selection of non-exposed cohort (★)	Ascertainment of exposure (★)	Outcome not present at start (★)	Comparability (max ★★)	Outcome assessment (★)	Follow-up long enough (★)	Adequacy of follow-up (★)	Total Score (max 9)
Liu SB, 2021	★	★	★	★	★	★	★	★	8
Genís C, 2016	★	★	★	★	★	★	★	★	8
Anger KE, 2010	★	★	★	★	★	★	★	★	8

Newcastle–Ottawa Scale (NOS) Criteria for Cohort Studies.

□ 1. Selection (Maximum 4 stars).

Representativeness of the exposed cohort.

★ Truly representative ICU patients (e.g., multicenter or consecutively enrolled patients).

☆ Selective or specific patient groups (e.g., single disease, single center, or non-consecutive enrollment).

Selection of the non-exposed cohort.

★ Drawn from the same community or population as the exposed group (e.g., same hospital or time period).

☆ Different source or not described.

Ascertainment of exposure.

★ Secure record (e.g., medical records, prescription databases) — objective assessment.

☆ Self-report, recall questionnaire — subjective assessment.

Demonstration that outcome of interest was not present at start of study.

★ Clearly stated that the outcome (e.g., AKI) was not present at baseline.

☆ Not stated or unclear.

□ 2. Comparability (Maximum 2 stars).

Comparability of cohorts on the basis of the design or analysis.

★ One star: The study controlled for the most important confounding factor (e.g., age, severity of illness).

★★ Two stars: The study controlled for two or more key confounders using multivariate regression, matching, or propensity score methods.

☆ No adjustment or only descriptive comparison.

□ 3. Outcome (Maximum 3 stars).

Assessment of outcome.

★ Outcome assessed objectively (e.g., laboratory-confirmed, blinded independent assessment).

☆ Subjective assessment or no description of assessment method.

Was follow-up long enough for outcomes to occur?

★ Yes (e.g., ICU outcomes, 28-day mortality, development of AKI, etc.).

☆ Inadequate duration or not reported.

Adequacy of follow-up of cohorts.

★ Complete follow-up or loss to follow-up <20%, or adequately explained.

☆ Loss to follow-up ≥20% with no explanation.

**Figure 4:**
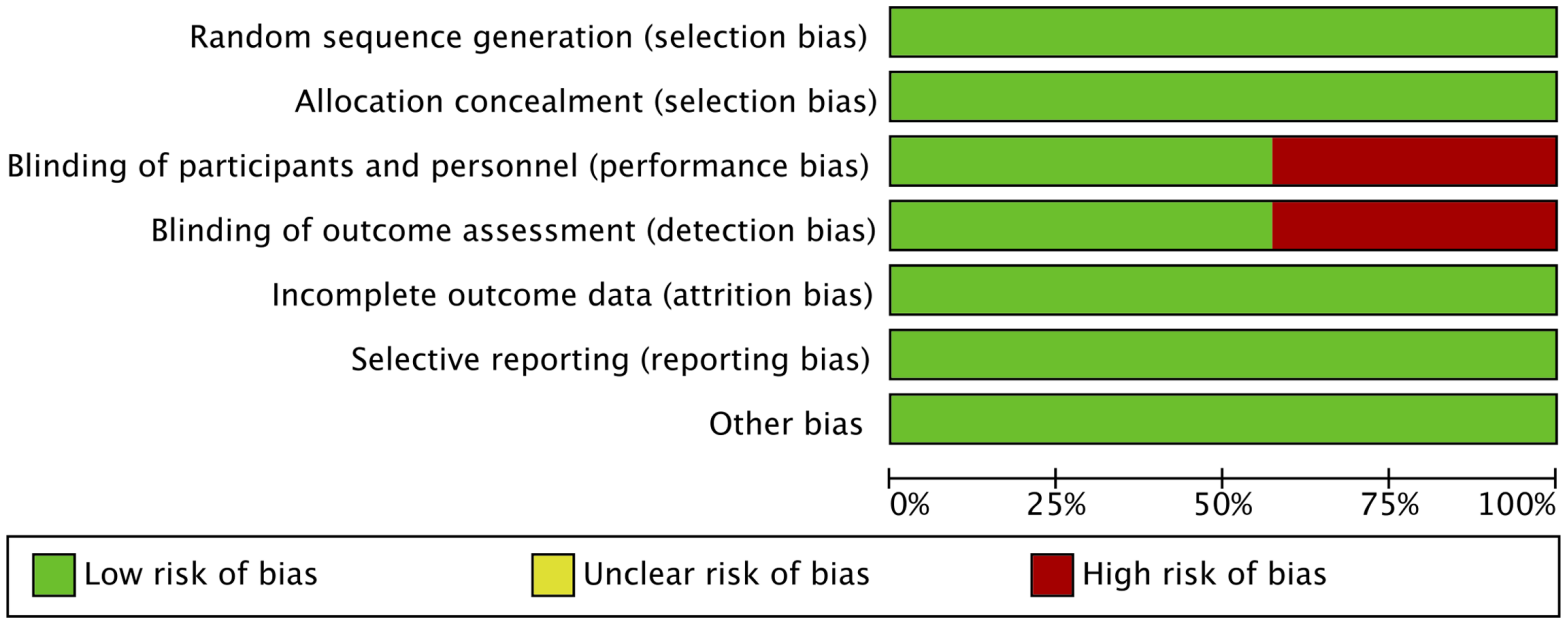
Detailed risk of bias analysis of the included trials. This figure presents the overall assessment of methodological quality across all included randomized controlled trials. Each domain of bias (e.g., random sequence generation, allocation concealment, blinding, incomplete outcome data, selective reporting, and other biases) is represented as a proportion of studies classified as low, unclear, or high risk of bias.

**Figure 5:**
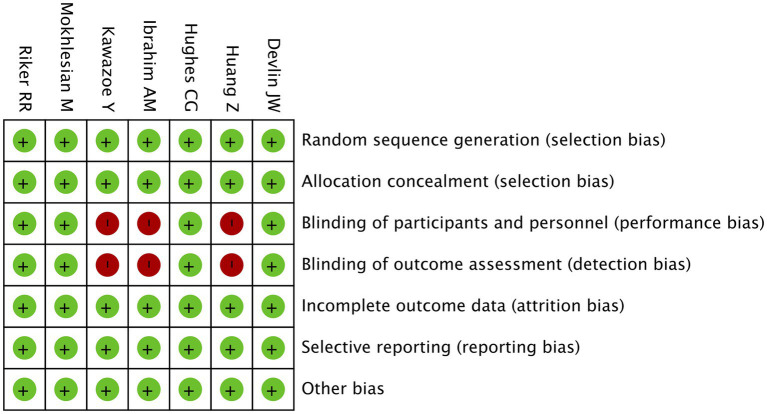
Risk of bias summary. This figure summarizes the risk of bias judgment for each included study. Each row represents an individual study, and each column corresponds to a specific domain of bias.

The risk of bias assessment for the included randomized controlled trials (RCTs) was conducted using the Cochrane RoB 2.0 tool, with results presented in both graphical and tabular formats. The risk of bias graph displays the distribution of bias across seven domains for all RCTs, showing the percentage of studies rated as low, unclear, or high risk of bias in each category (Figure 4). Key domains assessed include random sequence generation, allocation concealment, blinding (participants/ personnel and outcome assessment), incomplete outcome data, selective reporting, and other potential biases. The risk of bias summary table provides detailed ratings for each individual study (Devlin JW, Huang Z, Hughes CG, Ibrahim AM, Kawazoe Y, Mokhlesian M, Riker RR) across these same seven domains (Figure 5) (2, 16, 17, 19, 20, 22, 23). This allows for comparison of methodological quality between studies and identification of potential patterns in bias. The graphical representation indicates that most studies demonstrated low risk of bias in critical domains like random sequence generation and allocation concealment, suggesting generally robust randomization procedures. However, some studies showed higher risk in performance bias (blinding of participants/personnel) and detection bias (blinding of outcome assessment), which is common in pharmacological trials where complete blinding can be challenging. The summary table enables readers to quickly identify which specific studies may have higher risk in particular domains that could affect result interpretation.

## 4 Discussion

This meta-analysis comprehensively evaluated the safety profile of dexmedetomidine (DEX) sedation in adult ICU patients by synthesizing data from 10 clinical studies, including seven randomized controlled trials and three observational cohorts, with a total of 1,456 critically ill individuals. The key findings reveal that while dexmedetomidine does not significantly increase the overall risk of hypotension (OR = 1.15, 95% CI [0.91–1.47]), it is consistently associated with a significantly elevated risk of bradycardia (OR = 2.38, 95% CI [1.77–3.21]) and shows a modest but statistically significant increase in tachycardia (OR = 1.38, 95% CI [1.03–1.83]) with considerable heterogeneity (I^2^ = 93%). Among these outcomes, bradycardia emerged as the most robust and reproducible signal across studies, aligning with the drug’s known sympatholytic mechanism via selective *α*₂-adrenoceptor agonism. In contrast, the associations with hypotension and tachycardia were more variable and likely influenced by clinical context, such as underlying disease states, hemodynamic stability, and sedation protocols. These findings suggest that while DEX’s overall hemodynamic impact may be modest, certain high-risk populations—particularly elderly patients, those with sepsis, or those requiring vasopressors—may be more vulnerable to adverse effects.

The findings of this meta-analysis have direct clinical implications for the use of dexmedetomidine (DEX) in ICU sedation, particularly in relation to its cardiovascular safety profile. The most consistent and statistically robust result was the significantly increased risk of bradycardia (OR = 2.38, 95% CI [1.77–3.21], *p* < 0.00001; I^2^ = 0%), indicating a predictable pharmacodynamic effect related to DEX’s sympatholytic activity. This reinforces the necessity for continuous heart rate monitoring during DEX administration, especially in patients with baseline conduction disorders or those receiving concomitant AV node–blocking agents such as *β*-blockers. However, it is important to note that this same bradycardic effect, mediated by a reduction in excessive sympathetic tone, may be clinically desirable in specific patient populations. For instance, in patients with ischemic heart disease, DEX-induced heart rate control can reduce myocardial oxygen demand and improve supply, potentially offering a therapeutic advantage during periods of physiologic stress in the ICU. Nevertheless, harnessing this potential benefit while mitigating risks introduces significant clinical complexity. It necessitates intensive and multifaceted monitoring, including close tracking of hemodynamic status, heart rate and rhythm dynamics, and real-time electrocardiographic changes, to carefully titrate therapy. This sophisticated balancing act between therapeutic benefit and safety presents a considerable challenge in routine clinical practice. Therefore, further validation in well-designed prospective studies is essential to establish standardized monitoring protocols and precise patient selection criteria for the optimized use of DEX.

Although the pooled analysis did not show a statistically significant increase in hypotension risk (OR = 1.15, 95% CI [0.91–1.47], *p* = 0.25; I^2^ = 44%), notable outliers in subgroup analyses—such as Liu SB 2021 (OR = 3.26) and Huang Z 2012 (OR = 5.86)—suggest that elderly or septic patients, or those requiring vasopressor support, may still be at higher risk, warranting careful titration and close blood pressure surveillance during initiation. Post-marketing pharmacovigilance reports list hypotension in roughly 15–25% of DEX adverse events, particularly when loading boluses are used or when patients are septic (25, 26). In prospective trials, omission of bolus doses and proactive volume management appeared to neutralize this signal, which is consistent with the neutral risk seen in our pooled estimate and the only moderate between-study heterogeneity (I^2^ = 44%). By contrast, bradycardia emerged as the most prominent cardiac adverse effect, reflecting dexmedetomidine’s central sympatholytic action and underscoring the need for continuous ECG monitoring and judicious dose titration (26). The modest tachycardia signal may represent a compensatory response to vasodilation or a withdrawal phenomenon, meriting targeted mechanistic studies.

The association with tachycardia was modest but statistically significant (OR = 1.38, 95% CI [1.03–1.83]), though high heterogeneity (I^2^ = 93%) indicates that this effect is likely context-dependent. For example, Hughes CG 2021 reported increased tachycardia risk (OR = 3.14) in septic patients, whereas Riker RR 2009 observed a protective effect (OR = 0.43), highlighting the influence of population characteristics and baseline autonomic tone. Taken together, these results support the selective and individualized use of dexmedetomidine in ICU practice. Its favorable effects on ventilator synchrony, preservation of respiratory drive, and possible delirium prevention remain important advantages; however, its cardiovascular risks must be proactively managed through tailored dosing and structured hemodynamic monitoring to ensure patient safety while maximizing therapeutic benefit.

The landmark SPICE III trial in ventilated ICU patients found similar 90-day mortality between dexmedetomidine and usual care, with more bradycardia and hypotension in the dexmedetomidine arm (3). Two earlier JAMA trials also documented significant hemodynamic swings during prolonged infusions, highlighting the need for careful dosing and patient selection (4). A Bayesian re-analysis of SPICE III showed that age, rather than hypotension, modulated mortality, whereas a sepsis-specific meta-analysis suggested survival benefits at the cost of additional arrhythmias without extra hypotension (27, 28). Together, these data indicate that DEX safety is determined chiefly by heart-rate effects rather than sustained blood-pressure depression.

Besides, among the included studies, one utilized a combination of midazolam and an opioid as the control sedative. Sedation with dexmedetomidine was shown to attenuate inflammatory factors in septic shock and did not worsen hemodynamic parameters (16). In fact, the side effects associated with opioids, such as respiratory depression, nausea, vomiting, pruritus, ileus, and delirium, are notably prominent, whereas those associated with dexmedetomidine are comparatively minimal (29, 30). Therefore, a thorough evaluation of the respective risk–benefit profiles of opioids and dexmedetomidine is essential in clinical practice to facilitate informed choices.

Based on the comprehensive analysis presented, this meta-analysis has several important limitations that should be acknowledged. The primary constraints stem from the heterogeneity in study designs and outcome definitions across included trials. While the analysis incorporated both randomized controlled trials (RCTs) and observational studies (totaling 10 studies with 1,456 patients), significant variability existed in patient populations (e.g., sepsis vs. postoperative cardiac surgery), sedation protocols (monotherapy vs. adjunctive sedatives), and adverse event criteria (e.g., inconsistent thresholds for defining bradycardia or hypotension). This heterogeneity—particularly extreme for tachycardia outcomes (I^2^ = 93%)—complicates the generalizability of pooled estimates. Furthermore, the limited number of studies for specific outcomes (e.g., only 4 studies reporting tachycardia) and the small sample sizes of some trials (e.g., n = 33–48 in Huang 2012 and Devlin 2014) reduced statistical power to detect rare adverse events or confirm subgroup differences. The observational studies, though methodologically sound per NOS assessment, lacked rigorous adjustment for confounding variables (scoring only 1/2 in comparability), potentially biasing associations in these cohorts. Finally, the exclusion of non-English studies and reliance on published data may introduce selection bias, while the inability to perform dose–response analyses due to inconsistent dosing reporting limits practical guidance for risk mitigation. These limitations underscore the need for large, standardized RCTs focusing on high-risk subgroups and employing uniform adverse event definitions.

## Data Availability

The original contributions presented in the study are included in the article/Supplementary material, further inquiries can be directed to the corresponding author.
