# Supplementary material for: Safety and adverse events associated with dexmedetomidine for sedation in adult ICU patients: a systematic review and meta-analysis
Source: Front Med (Lausanne). 2025 Nov 7;12:1677955. doi: 10.3389/fmed.2025.1677955 (PMC12634627; doi:10.3389/fmed.2025.1677955)
Supplement: Supplementary file 1 [file Table_1.docx]

**Supplementary Table 1. The detailed search terms in database of PubMed, Embase, and the Cochrane Library.**

| **Database** | **Search term** |
| --- | --- |
| **PubMed** | ("dexmedetomidine"[MeSH Terms] OR "dexmedetomidine"[All Fields] OR "right medetomidine" OR "Precedex")  AND  ("intensive care units"[MeSH Terms] OR "ICU"[All Fields] OR "critical care"[All Fields] OR "intensive care"[All Fields])  AND  ("sedation"[MeSH Terms] OR "sedation"[All Fields])  AND  ("adult"[MeSH Terms] OR "adult"[All Fields] OR "aged"[MeSH Terms] OR "aged"[All Fields] OR "18 years"[All Fields] OR "age 18"[All Fields])  AND  ("adverse effects"[MeSH Terms] OR "adverse event"[All Fields] OR "side effect"[All Fields] OR "toxicity"[All Fields] OR "safety"[All Fields])  NOT  ("case reports"[Publication Type] OR "case report"[Title] OR "case series"[Title])  NOT  ("letter"[Publication Type] OR "editorial"[Publication Type] OR "comment"[Publication Type] OR "correspondence"[Title])  NOT  ("review"[Publication Type] OR "meta-analysis"[Publication Type])  NOT  ("conference abstract"[Publication Type] OR "conference paper"[Publication Type])  NOT  ("protocol"[Title] OR "study protocol"[Title]) |
| **EMBASE** | ('dexmedetomidine'/exp OR dexmedetomidine:ti,ab OR precedex:ti,ab OR 'right medetomidine':ti,ab)  AND  ('intensive care unit'/exp OR 'critical care'/exp OR 'intensive care':ti,ab OR ICU:ti,ab)  AND  ('sedation'/exp OR sedation:ti,ab)  AND  ('adverse drug reaction'/exp OR 'side effect'/exp OR 'toxicity'/exp OR 'safety'/exp OR adverse event*:ti,ab OR side effect*:ti,ab OR toxicity:ti,ab OR safety:ti,ab)  AND  ('adult'/exp OR adult*:ti,ab OR 'aged'/exp OR aged:ti,ab)  AND  [humans]/lim AND [article]/lim  NOT  ('case report'/exp OR 'case series':ti OR 'letter'/exp OR 'editorial'/exp OR 'comment'/exp OR 'correspondence'/exp OR 'conference abstract'/it OR 'review'/exp OR 'meta analysis'/exp OR 'study protocol'/exp OR protocol:ti) |
| **Cochrane Library** | ("dexmedetomidine":ti,ab,kw OR "right medetomidine":ti,ab,kw OR "Precedex":ti,ab,kw)  AND  ("intensive care":ti,ab,kw OR "ICU":ti,ab,kw OR "critical care":ti,ab,kw)  AND  ("sedation":ti,ab,kw)  AND  ("adult":ti,ab,kw OR "aged":ti,ab,kw OR "18 years":ti,ab,kw OR "age 18":ti,ab,kw)  AND  ("adverse effects":ti,ab,kw OR "adverse event":ti,ab,kw OR "side effect":ti,ab,kw OR "toxicity":ti,ab,kw OR "safety":ti,ab,kw)  NOT  ("case report":ti OR "case reports":ti OR "case series":ti)  NOT  ("letter":pt OR "editorial":pt OR "comment":pt OR "correspondence":ti)  NOT  ("review":pt OR "meta-analysis":pt)  NOT  ("conference abstract":pt OR "conference paper":pt)  NOT  ("protocol":ti OR "study protocol":ti) |
