# Supplementary material for: Safety and adverse events associated with dexmedetomidine for sedation in adult ICU patients: a systematic review and meta-analysis
Source: Front Med (Lausanne). 2025 Nov 7;12:1677955. doi: 10.3389/fmed.2025.1677955 (PMC12634627; doi:10.3389/fmed.2025.1677955)
Supplement: Supplementary file 2 [file Supplementary_file_1.docx]

**Supplementary Figure 1. Flowchart of studies selection.**
